# Preliminary Study on the Echo-Assisted Intersphincteric Autologous Microfragmented Adipose Tissue Injection to Control Fecal Incontinence in Children Operated for Anorectal Malformations

**DOI:** 10.3390/children7100181

**Published:** 2020-10-13

**Authors:** Giovanni Parente, Valentina Pinto, Neil Di Salvo, Simone D’Antonio, Michele Libri, Tommaso Gargano, Vincenzo Davide Catania, Giovanni Ruggeri, Mario Lima

**Affiliations:** 1 Pediatric Surgery Department, Sant’Orsola-Malpighi University Hospital, 40138 Bologna, Italy; neildisalvo@hotmail.com (N.D.S.); alcmeone1@libero.it (S.D.); mlibri31@yahoo.it (M.L.); tommaso.gargano2@unibo.it (T.G.); vdcatania1985@gmail.com (V.D.C.); giovanniruggeri9@gmail.com (G.R.); mario.lima@unibo.it (M.L.); 2 Plastic Surgery, Sant’Orsola-Malpighi University Hospital, 40138 Bologna, Italy; valentina.pinto@aosp.bo.it

**Keywords:** anorectal malformation, fecal incontinence, bowel management, endoanal ultrasound, anal-lipofilling

## Abstract

Aim of the study: To assess the efficacy of a novel technique (echo-assisted intersphincteric autologous microfragmented adipose tissue injection, also called “anal-lipofilling”) in the management of non-responsive fecal incontinence in children born with anorectal malformations (ARMs). Methods: Following ethical committee approval (CHPED-MAR-18-02), anal-lipofilling was proposed to patients with fecal incontinence not responsive to medications or bowel management (bowel enema and/or transanal irrigation automatic systems), then a prospective study was conducted. Anal-lipofilling consisted of three phases: lipoaspiration from the abdominal wall, processing of the lipoaspirate with a Lipogems system and intersphincteric injection of the processed fat tissue via endosonographic assistance. A questionnaire based on Krickenbeck’s scale (KS) was administered to the patients to evaluate the clinical outcome. Main Results: Four male patients (three recto-urethral fistula, and one recto-perineal fistula) underwent the anal-lipofilling procedure at a mean age of 13.0 ± 4.2 yrs. There were no complications during or after the procedure. From an initial assessment of the patients there was an improvement in the bowel function at a median follow up of 6 months, with better scores at KS (100% Soiling grade three pre-treatment vs. 75% grade one post-treatment). Conclusions: Even if our Study is preliminary, echo-assisted anal-lipofilling could be considered as a feasible and safe alternative technique in the management of the fecal incontinence in non-responding ARMs patients. More studies are still necessary to support the validity of the implant of autologous adipose tissue in the anal sphincter as a therapy for fecal incontinence in children born with ARMs.

## 1. Introduction

The management of fecal incontinence is one of the most challenging aspects in the follow-up of patients with anorectal malformation (ARM) after undergoing surgery. Incontinence affects these patients’ social acceptance and therefore determines enormous repercussions on their psychology [1].

The instruments the surgeon can count on have not improved in recent years and are essentially based on the use of regular enemas and osmotic laxatives.

Recently, in other disciplines like plastic surgery and orthopedic surgery, the use of adipose tissue has taken place in the repair of tissue damage [2,3,4,5,6].

Some authors have tried the use of an autologous fat graft in treating adults’ fecal incontinence and reported positive results of their preliminary experience [7,8].

This background led us to think about a possible application in the management of incontinence in our type of patients.

Therefore, we treated four patients operated on for ARM in our center suffering from fecal incontinence with echo-assisted autologous intersphincteric injection of autologous microfragmented adipose tissue (anal-lipofilling).

In addition, considering our increasing experience on endoanal ultrasonography, such injections were performed via endoanal-US guidance to guarantee a more precise transplantation site.

We recorded a preliminary result of this technique in order to decide whether or not it can take place in the follow up of these patients.

## 2. Materials and Methods

A retrospective study was conducted. All patients operated on for ARM, suffering from fecal incontinence with no or poor benefit from bowel management and treated in our center with echo-assisted anal-lipofilling were considered but those with less than 6-months follow-up were excluded from the study (ethical committee approval CHPED-MAR-18-02).

To the selected patients were administered a questionnaire about fecal incontinence before and after every procedure of anal-lipofilling; fecal incontinence was evaluated with Krickenbeck’s scale (KS) [4] that is composed of four items (Table 1):

1. Voluntary bowel movements (or the ability of patients to feel and hold the defecatory stimulus) recorded as “absent” (indicated as 1.0) or “present” (1.1);

2. Soiling that, if present, is stratified in three grades: once or twice per week (2.1), every day with no social problems (2.2) and every day with social problems (2.3). Absence of soiling will be indicated as 2.0;

3. Constipation that, if present, is divided too in three grades: manageable with changes in diet (3.1), requires laxatives (3.2), resistant to laxatives and diet (3.3). Absence of constipation will be reported as 3.0.

Questionnaire recorded any changes in frequencies of bowel management.

**Figure 1:**
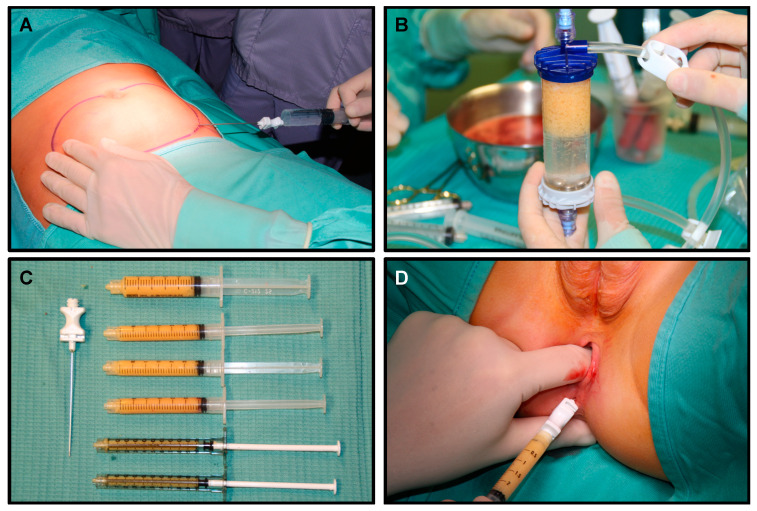
Lipofilling procedure: (**A**) Lipoaspiration, (**B**) Adipose tissue processing technique, (**C**) Adipose tissue ready for injection; (**D**) intersphincteric adipose tissue injection.

Anal-lipofilling technique: The patient is positioned supine; in our experience, general anesthesia is required. Prophylactic antibiotics are given intravenously prior to skin incision. An 11-blade scalpel is used to make a stab incision in the subcutaneous tissue. A solution of Epinephrine 2 mcg/mL is injected subcutaneously using an 18-Gauge cannula followed by 10 min resting period to allow an adequate emulsification. The adipose tissue is then harvested with the lipo-suction cannula connected to a 10 mL Vaclok syringe (Figure 1A). This syringe allows the plunger to lock, creating a negative pressure vacuum. Liposuction of abdominal fat is performed using brisk broad strokes until a total of 30 to 60 mL of aspirate is obtained depending on the patient’s body habitus. With each successive liposuction attempt, the Vaclok syringes become pinker in color, indicating a more hemorrhagic aspirate with less adipose tissue yield. The adipose tissue from each of the Vaclok syringes is transferred to a single 20 mL syringe using a luer lock connector. 

**Figure 2:**
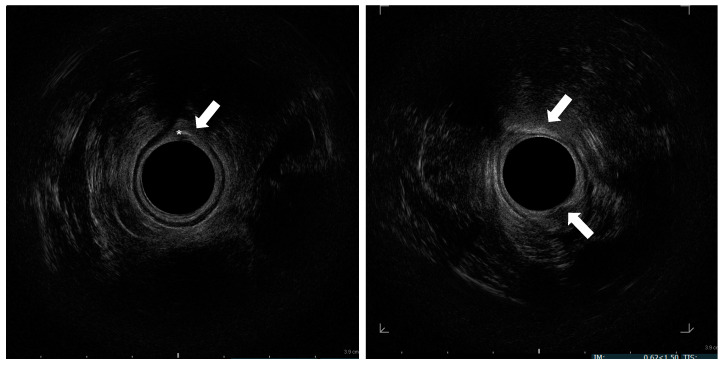
Endosonographic axial sections of the middle anal canal. Asterisk: needle in the internal anal sphincter; Arrow: injected fat tissue h 6 and 12.

The processing cylinder (Lipogems^®^) containing 5 stainless steel marbles is connected to 2 hoses. The hose attached to the blue size reduction filter is connected to a bag of normal saline. The gray size reduction filter on the opposite end of the cylinder is attached to a hose connected to a waste bag, which rests on the floor. The cylinder is then rotated so that the gray filter points upward. Both the blue filter hose and waste hose are opened to allow the cylinder to fill with normal saline while holding it vertically and shaking intermittently to remove air bubbles. Both the hoses are then clamped closed once the cylinder is filled completely. The 20 mL syringe containing the lipoaspirate is connected to the blue filter. The adipose tissue is injected into the processing cylinder through the blue filter with the clamp on the gray waste side open. With the blue filter pointing up, both clamps are opened to allow elimination of blood and oily impurities into the waste bag. Once the fluid is transparent, the shaking process will begin. Both the hoses are clamped closed, and the cylinder is shaken for 30 s to allow the action of the steel spheres to emulsify and microfracture the adipose tissue (Figure 1B). This sequence is repeated followed by a final wash. Two 10 mL luer lock syringes are connected to both sides of the chamber in order to allow removal of the stem cell and adipose tissue product. The processing cylinder is flipped with the gray filter at the top. The blue filter hose is opened. Through the syringe connected to the blue filter a full 10 mL of saline is drawn. The blue filter hose is closed. The cylinder is held vertically with the gray filter at eye level. Acting on the syringe connected to the blue filter, the saline is pushed into the cylinder, thus forcing the stem cell and adipose tissue product through the gray filter into the empty syringe connected on top. The top syringe containing the stem cells is removed and placed facing up to allow the cells to settle within the syringe and separate from the excess fluid. The cylinder is shaken slightly, and the process is repeated to continue retrieving the cells. This is performed until no more cells are yielded from the cylinder. The goal is to obtain 30 mL of final stem cell product (Figure 1C). At this point, with the patient in the gynecological position, three injections of 10 mL of adipose tissue are performed at three out of four quadrants of middle anal canal under endoanal-US guidance (Figure 1D), precisely in the intersphincteric plane (Figure 2).

As regards the echo-assistance, we used an anorectal 3D 2052 transducer (17 mm diameter, 13 MHz) with a BK-medical flexfocus 800 US system (BK Medical Italia srl, Melegnano, 20077, Milan, Italy).

The aspiration and the processing of the adipose tissue was performed by a plastic surgeon of our institute while the injection by a pediatric surgeon of our equip.

Possible described complications of this procedure could be visible irregularities of the lipoaspiration site (in our series the abdominal wall), seromas, local infection and extremely rare major complications such as fat embolism syndrome (risk extremely low considering the small size of the cannula used to lipoaspirate) and extended infection.

Patients that underwent echo-assisted anal-lipofilling were discharged the day after the procedure.

## 3. Results

We treated four patients with intersphincteric injection of autologous adipose tissue (anal-lipofilling). The patients were all male, three recto-urethral fistulas (75%), and one recto-perineal fistula (25%) and underwent the first anal-lipofilling at a mean age of 13 ± 4.2 yrs (range: 8–17 yrs). All patients underwent colonostomy open at birth, postero-sagittal anorettoplasty (PSARP) between 2 and 4 months of life and colonostomy closure about a month after the PSARP.

All four patients suffered of total fecal incontinence and presented the same score at Krickenbeck’s scale: 1.0, 2.3, 3.0 and needed an enema every day to help bowel control.

The total number of procedures performed was nine and the mean number of procedures per patient was 2.0 ± 1.3 (range: 1–4).

**Figure 3:**
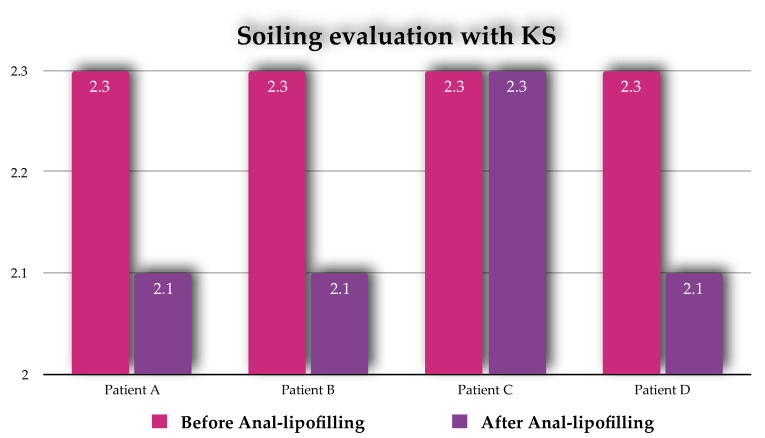
Results of Anal-Lipofilling on Soiling reported as Krickenbeck scale (KS) score.

Three patients treated (75%) showed a significant improvement in continence with a KS score of 1.0, 2.1, 3.0 after every single procedure and a reduction in the number of enemas from once daily to one or two weekly (Figure 3).

One patient (25%) did not benefit from the anal-lipofilling, no changes on the KS recorded before and after the procedure (Figure 3).

The mean interval between the procedures in the ones who underwent more than one anal-lipofilling was 343.8 ± 220.1 days (range 203–733 days); the re-do was determined by the progressive loss of efficacy of the previous procedure.

No complications were recorded during and after the procedure.

## 4. Discussion

Fecal incontinence is a major problem in ARM management after surgical correction and the older is the patient the more important becomes the follow up due to social and psychological repercussions [9].

Nowadays bowel management is the assessed and only way to help these patients to control their bowel function, but it is an invasive and not always tolerable procedure [10,11]. The efforts of pediatric surgeons should be focused on find out new ways to achieve a good bowel control with less invasive methods.

Some authors have tried the use of autologous fat graft in treating adults’ fecal incontinence and reported positive results of their preliminary experience.

For instance, Gentile M. et al. [7] observed an increase in resting pressure (by at least 10 mmHg) and thickness of the interal anal sphincter, respectively, at ano-rectal manometry and by US evaluation at the sixth months follow up.

Additionally, Jeong H. et al. [8] compared results of anolipofilling with pre- and post-operative ano-rectal manometry and endosonography.

According to preliminary results of this procedure in adults’ incontinence reported in the literature and the wide use of autologous adipose tissue transplant in other disciplines, we decided to apply this technique to pediatric patients affected by ARM.

We performed a total of nine lipofilling procedures from 2016 and we recorded a preliminary result from the follow up and the parent’s opinions.

In three patients out of four treated, anal-lipofilling improved the continence with less episodes of loss of bowel contents, a decrease in number of enemas (KS: from 1.0, 2.3, 3.0 to 1.0, 2.1, 3.0 after the procedure) and parents were extremely satisfied of the results.

From the KS scores, it seems that the procedure does not influence the ability of patient to feel the urge to defecate, but since this disorder could be related to neurological impairment of sacral fibers due to the ARM itself, it is reasonable it could not be solved by an injection of adipose tissue.

It is difficult at this moment in time to have a certain explanation of the way this procedure works for fecal continence. A valid explanation could be that the injections, due to a mass-effect, helps the continence of the anal sphincter increase the pressure needed for defecation and so reducing the episodes of fecal loss, thus acting as a bulking agent.

Furthermore, it has been postulated that adipose staminal cells maybe somehow involved in increasing the amount of muscle cells of the anal sphincter thanks to their differentiation potential, but it is not currently documentable in our population of patients.

Endoanal-US guidance has never been used in this procedure and we believe it could be of some advantage thanks to the precise site of injection—that is the intersphincteric plane.

The anal-lipofilling seems to have a time-related efficacy that could be explained by the partial reabsorption of the adipose tissue injected; considering that when the parents came back to our institution to report a reduction in the effects we shortly after programmed and performed a new one, we could assume the average interval between procedures as the average duration of the effects of the anal-lipofilling (in our cohort of study: 343.8 ± 220.1 days).

No complications were recorded.

We are aware our results are limited to the paucity of patients enrolled.

Nevertheless, our preliminary outcomes encourage us to continue proposing this novel technique to our patients.

It is necessary to understand if the anal-lipofilling works better on secondary overflow incontinence or on true incontinence due to intrinsic sphincteric deficiency in order to improve patients’ selection.

Moreover, the next step of our study will include both pre- and post-operative ano-rectal manometry evaluation.

## 5. Conclusions

Our preliminary results on the anal-lipofilling technique are encouraging—75% of patients treated improved their continence with less episodes of involuntary fecal loss with no complications recorded.

More studies are needed to assess the exact mechanism of action, to better evaluate the duration of the effects and, therefore, define an adequate follow-up.

## Figures and Tables

**Table 1:** International classification (Krickenbeck) for postoperative results.

**1. Voluntary Bowel Movements**
Feeling of urge, capacity to verbalize and hold the bowel movements 1.0. No. 1.1. Yes.
**2. Soiling/Fecal Incontinence**
2.0. No. 2.1. Occasionally (once/twice a week). 2.2. Every day, no social problem. 2.3. Constant, social problem.
**3. Constipation**
3.0. No. 3.1. Manageable with diet. 3.2. Requires laxatives. 3.3. Resistant to diet and laxatives.
